# Editorial: CNS Recovery after Structural and / or Physiological / Psychological Damage

**DOI:** 10.3389/fncel.2016.00225

**Published:** 2016-10-06

**Authors:** Marie Z. Moftah, Emmanuel Moyse

**Affiliations:** ^1^Neuroplasticity and Pain Laboratory, Zoology Department, Alexandria UniversityAlexandria, Egypt; ^2^Unité PRC, INRA de Tours, François Rabelais UniversityTours, France

**Keywords:** CNS recovery, structural damage, physiological damage, psychological damage, CNS

The central nervous system (CNS) is critically vulnerable to damage in post-natal and adult life, for two reasons. First, its major constitutive cell type, neuron, is more fragile than most other differentiated cell types, due to its exclusive dependency on glucose for energetic metabolism, to its high chronic demand of oxygen supply, to lower levels of antioxidant defenses and to extreme structural vulnerability of its long and thin axonal expansions. Second, any neuron damage or loss has dysfunctional outcome because of the specific dependency of nervous functions on the topography of neuronal interconnections. CNS damage can occur from environmental threats including both physical (injuries) and psychosocial (stress) risks. Consequently, several forms of brain plasticity can be affected and trigger adaptive responses to maintain homeostasis or functional recovery. These processes engage the immune system, the autonomic nervous system (ANS) besides the hypothalamo-hypophyseo-adrenal (HPA) axis via specific CNS-borne neurotransmitters, hormones, neuropeptides and growth factors. The goal of this Research Topic is to review the cellular and molecular mechanisms of damageinduced CNS plasticity through a selection of original research articles in this field. Derghal et al. use the neuroendocrine regulation of food intake to document the adaptive role of a recently emerged mechanism of neuroplasticity: neuronal synthesis of microRNAs (miRNAs) i.e., short non-coding RNA molecules that repress gene expression at the post-transcriptional level by binding to target mRNAs. They screened *in silico* the brain gene target of the major anorexigenic hormone leptin, POMC, for miRNA binding sites. It revealed 3 candidate miRNAs, which were indeed found upregulated in the hypothalamus of congenitally obese, leptin-deficient ob/ob mouse. This result provides a new mechanism of hormone-dependent neuronal plasticity with relevance to a physio-pathological adaptation. Segura et al. also use leptin hormonal signaling in the cerebral regulation of food intake, to document another effector mechanism of neuroplasticity: modulation of neurogenesis from adult neural stem cells. They report that leptin *in vitro* depresses adult neurogenesis from the canonical neural stem cells of the rodent subventricular zone (SVZ) through Ob-receptor-induction of apoptosis in immunocytochemically identified neuronal progenitors. This hormone-induced neurotoxicity is shown to be mediated through the signaling pathway of extracellular signal-regulated kinases ERK-1/2 and cyclin D1, i.e., the molecular switch between cell division and apoptosis. Zaky and Moftah address post-lesional induction of neurogenesis-stimulating molecules in the spinal cord of the Amphibian Pleurodeles. This animal model displays extensive neural regeneration including both structural and locomotor restorations. They report post-lesional *in vivo* inductions of FGF2, i.e., the major intercellular mitogen for adult neural stem cells, and of the stem cell marker nestin. These data provide cues for post-lesional sequelae curing in adult mammals, especially via cellular therapy. Kilic et al. characterize motor function and histological markers of brain plasticity following stroke induction by middle cerebral artery occlusion in adult mouse, treated or not with the secondary stroke-preventing clinical drug HMG-CoA reductase inhibitor rosuvastatin. They show rosuvastatin treatment increases functional motor recovery, neuronal survival and capillary density and decreases forebrain atrophy as compared to untreated lesioned mice. A single molecule-targeted drug can thus help neurological recovery via lesion-induced neuroplasticity potentiation. Zaky et al. use bacterial lipopolysaccharide (LPS)-induced neuroinflammation in adult rats as an *in vivo* model to investigate the mechanisms of neuroprotection by the drugs valproic acid (inhibitor of the epigenetically acting histone deacetylase-1) and curcumin. Strong synergy of the two drugs were shown by *in vivo* combination-induced additivity of their respective effects on histological and molecular markers of neuroinflammation, on biochemical markers of LPS-induced oxidative stress, and on LPS-induced repression of the five members of Let-7 miRNA family. The combined drugs suppressed LPS-induced neuroinflammation and restored oxidation marker, antioxidant defense and Let-7 miRNA to their control levels. Khalil et al. designed a computational assay to investigate the potential use of a recent cognitive psychological test of associative learning capacity (Acquired Equivalence Associative Learning Task, AEALT) to assess cognitive impairment of the Generalized Tonic Clonic (GTC) epilepsy in human clinics. Test application on a small cohort of GTC epileptic and control age- matched subjects confirmed the previously reported functional connectivity between hippocampus and basal ganglia, which validates the computational approach of this pathological brain plasticity. Najimi et al. provide a detailed mapping of the neuropeptide neurotensin high affinity receptors in the neonatal human hypothalamus and its evolution during the first year of age. They thus document developmental plasticity of the brain, reporting in particular strikingly higher densities of neurotensin receptors in the infant posterior hypothalamus than in adult, and a density decrease in the preoptic area during the first neonatal year. Chigr et al. report a differential effect of acute stress on neurotransmitter expression in the two brain centers of food intake regulation in adult rat. In either center, anorexigenic neuropeptide expression is up-regulated first and followed by delayed upregulation of orexigenic neuropeptides, which accounts for stress-induced anorexia. This phenotypical plasticity occurs earlier in the brainstem satiety center than in the long-term modulatory hypothalamus. El Marzouki et al. investigate the effects of repeated cold stress on spatial learning and memory in adult rat. Daily behavioral evaluation of learning and memory was combined in all experimental rats with electrophysiological assay of hippocampal LTP at the end of the 5-day-peiod of repeated stress. Gender-differential impacts of stress on brain plasticity were thus characterized. Mahmoud et al. provide the first mapping of nitric oxide (NO)-producing neurons in Urodeles' spinal cord, indicating their involvement in the dually terrestrial-aquatic locomotion of Salamanders. NO is a short-lived gaseous neurotransmitter, which is involved in mammalian plasticity and in brain development. Salamander with its bimodal locomotion is a precious model for the mechanisms underlying the developmental plasticity of vertebrate locomotion.

## Author contributions

All authors listed, have made substantial, direct and intellectual contribution to the work, and approved it for publication.

### Conflict of interest statement

The authors declare that the research was conducted in the absence of any commercial or financial relationships that could be construed as a potential conflict of interest.

